# Eco-Friendly Pavements Manufactured from Mixed Recycled Aggregates Obtained from Construction and Demolition Waste: An Industrial-Scale Validation

**DOI:** 10.3390/ma16247544

**Published:** 2023-12-07

**Authors:** Manuel Contreras-Llanes, Manuel Jesús Gázquez, Maximina Romero

**Affiliations:** 1 Department of Sociology, Social Work and Public Health, Research Centre for Natural Resources, Health and Environment (RENSMA), University of Huelva, 21007 Huelva, Spain; 2 Department of Applied Physics, Marine Research Institute (INMAR), University of Cádiz, 11510 Cádiz, Spain; manueljesus.gazquez@uca.es; 3 Department of Materials, Eduardo Torroja Institute for Construction Sciences (IETcc-CSIC), 28033 Madrid, Spain; nromero@ietcc.csic.es

**Keywords:** concrete paver blocks, concrete kerb units, recycling, construction and demolition waste, recycled aggregates

## Abstract

This study aimed to validate that laboratory-scale results could be commercially replicated when manufacturing marketable precast concrete. Construction and demolition waste (CDW) was separated into two (fine and coarse) recycled aggregates (RAs). Precast paver and kerb units were fabricated by partial or total substitution of natural aggregates (NAs) by RAs. The study involved the comprehensive characterisation of raw materials, including particle size distribution, mineral composition, and elemental composition. Paver blocks and kerbs manufactured with up to 50% RAs showed mechanical resistance (T = 3.7 ± 0.2 and B = 5.3 ± 0.6 MPa, respectively), water absorption between 5.3–5.7%, and abrasion resistance (approximately 20.2 mm), which met the standard requirements (UNE-EN 1340:2004 and UNE-EN 1338:2004). Furthermore, industrial-scale precast pavement units demonstrated strength and durability suitable for heavy traffic areas. A reduction of 13% in cement content could maintain the requirements with a partial RA substitution of 25%, offering economic and environmental benefits. Therefore, it is feasible at an industrial level to replace NAs with RAs, promoting durability and technological properties with a positive environmental impact and considerably reducing CO_2_ emissions by up to 65%. Overall, pavers with RAs manufactured at the laboratory scale met mechanical standards, and the kerb stones showed improvements in abrasion resistance. On an industrial scale, kerb stones and precast blocks with specific substitutions can meet strength, water absorption, and abrasion requirements, allowing a reduction in cement content.

## 1. Introduction

Waste valorisation is currently growing, driven by EU policies aiming to promote the circular economy concept by efficiently using raw materials and reducing waste disposal. To this end, waste management strategies must prioritise the regulatory hierarchy of prevention, reduction, reuse, and recycling [1]. Proper waste valorisation not only offers environmental benefits but also serves as an excellent alternative to traditional waste management methods, helping to achieve a sustainable circular economy [2].

This direction in waste management, especially using construction materials, holds the potential to generate new products with considerable added value. These products can compete with traditional materials [3,4,5,6] and be used in the development of new materials for specific applications [7,8,9,10]. In addition, the environmental benefits of efficient waste valorisation must be recognised, as it presents a sustainable solution to the waste management challenge [11].

Among the wide range of waste streams worldwide, the magnitude of construction and demolition waste (CDW) in the European Union stands out, accounting for approximately one-third of all waste generated [12]. CDW comprises diverse materials, including concrete, gypsum, wood, bricks, metals, plastic, glass, and solvents, most of which are recyclable. Establishing a protocol for the proper management of these wastes is essential to ensure correct component separation [13]. In this sense, the Waste Directive 2018/851[1] appointed the goal of recycling 70% of CDW by 2020. However, although a few countries, such as Denmark and Germany, have developed and implemented rules allowing the recycling of up to 90% of CDW, the EU’s average recycling rate for this waste remains approximately 50% [12].

In recent studies, the U.S. Environmental Protection Agency (EPA) estimated that the volume of CDW generated in the United States is twice the amount of municipal solid waste (MSW), with a reuse and recycling rate ranging from 15 to 20% [14]. Similar rates were observed in other countries, such as Japan, Korea, and Singapore [15]. In contrast, China generated approximately 30–40% of its waste as CDW, but the reuse and recycling rate was considerably lower, at only 5% [16].

CDW has been extensively studied for different applications as a substitute for natural aggregates (NAs), including the production of glassy materials [17], pavement construction [18,19], the manufacture of hydraulic concrete [20,21,22,23,24,25,26,27], unbound subbase mixtures [28,29], use as a filler in asphalt mixtures [30], and brick [31,32] and concrete block [33,34] production. The substitution of NAs is an important aspect because the United Nations Environment Programme (UNEP) estimated that 40–50 billion metric tons of sand, gravel, crushed stone, and aggregates have been extracted from the global environment [35], suggesting that the amount of extracted material could exceed the planet’s natural replenishment rates. For that reason, a primary goal in the valorisation of CDW, especially in construction materials, is the future commercial-scale use of the new materials obtained, substituting the use of NAs by CDW to decisively contribute to a “circular economy” as a main pillar of sustainable development. However, achieving this goal will be challenging. In this context, two main obstacles impeding extensive CDW recycling have been identified. The first concern is the uncertainty regarding potential health risks for workers using these materials. The second issue is the lack of confidence in the quality of CDW materials. These factors have substantially influenced the demand for CDW recycling, often slowing or even halting the valorisation of this waste [12,13].

In a recent study, the authors reported that adding up to 50 wt.% of fine recycled aggregates (FRA) obtained from CDW or incorporating 25 wt.% FRA and coarse recycled aggregates (CRA) in concrete manufacturing yielded properties similar to reference materials made only with NAs [32]. Additionally, laboratory-scale paving blocks and kerb units containing these recycled aggregates (RAs) were fabricated [36,37], successfully meeting the requirements specified in the UNE-EN 1338 and UNE-EN 1040 standards [38,39]. Values of water absorption lower than 6% and tensile strength higher than 3.6 MPa were achieved, showing that the use of RAs in percentages of up to 50 wt.% was feasible. However, the bulk density and compressive strength declined by a factor of 1.5 when NAs were substituted for RAs at percentages above 50%.

In this context, the main objective of this study was to prove that successful laboratory-scale outcomes were transferrable to the industrial-scale manufacture of precast concrete paving units, encompassing paving blocks and kerb stones, with strong commercial viability. Furthermore, this approach could not only enhance the technical properties of these materials beyond conventional options but also deliver a commendable environmental footprint. This study extensively examined RAs, including their particle size, mineral composition, and elemental content. The research also analysed the properties of concrete specimens and precast pavement units made with varying levels of RAs, focusing on water absorption, porosity, density, and strength. Additionally, industrial-scale precast units were analysed for mechanical performance, abrasion resistance, freeze-thaw durability, and cement content reduction. The environmental and economic benefits, such as reduced CO_2_ emissions, raw material demand, and cement consumption, associated with incorporating RAs in precast pavement, as compared to NAs, were also assessed.

## 2. Materials and Methods

### 2.1. Materials and Sample Preparation

**Figure 1:**
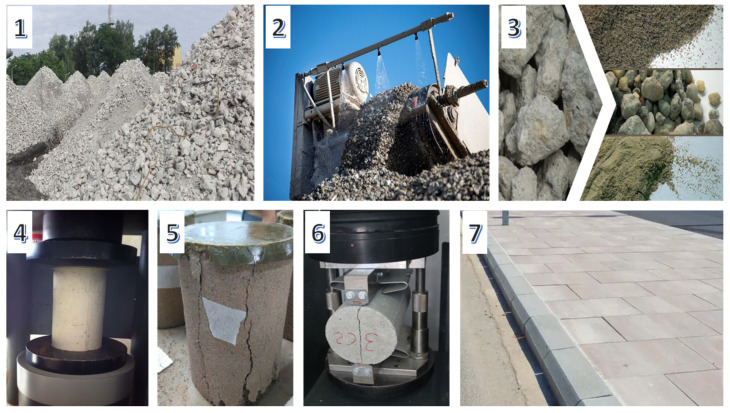
General procedure. (**1**) CDW selection. (**2**) CDW pretreatment. (**3**) Recycled aggregates. (**4**) Concrete testing specimen. (**5**) Characteristic prismatic fracture after compressive strength test. (**6**) Characteristic longitudinal fracture after tensile-splitting-strength method. (**7**) Industrial precast concrete pavement.

The general procedure of this research is summarised in Figure 1 and was divided into three major group stages: (1) CDW pretreatment; (2) Property testing (laboratory scales); and (3) Industrial scale validation.

#### 2.1.1. Materials

In this study, several samples of inorganic CDW fractions were examined. The materials were supplied by a management plant located in the province of Murcia, Spain, situated only 50 km from the precast company “Montalbán y Rodríguez S.L.”, also based in the region of Murcia, which supplied other materials, such as RAs, sand, gravel, cement, and additives, used in this research.

Previous studies revealed differences between NAs and CDW [21,22,32,40,41]. NAs tend to exhibit consistent quality and uniformity in terms of size and physical properties, while recycled aggregates from CDW can vary in quality and size due to the diversity of construction materials included. Consequently, a method was implemented to separate the most resistant and suitable RAs through mechanical treatment, involving crushing, grinding, sieving, and impurity removal of the CDW [32,36,37]. This process yielded two distinct RA fractions: FRA with particle sizes < 4.8 mm and CRA with particle sizes > 4.8 mm.

#### 2.1.2. Laboratory-Scale Stage

RAs (FRA < 4.8 mm and CRA > 4.8 mm) were dried in the laboratory at 105 °C for 24 h to eliminate moisture. Subsequently, different concrete mixtures (as listed in Table 1) were prepared using RAs as substitutes for NAs (sand and gravel) at different proportions (0, 25, 50, 75, and 100 wt.% FRA and/or CRA). Ordinary Portland cement (OPC) type I with a compressive strength of 32.5 N·mm^−2^ was used. Moreover, a high-activity water-reducing/superplasticiser additive based on polycarboxylates, specifically MasterCast 731 prescribed by the BASF company technical department (Ludwigshafen, Germany), was employed to enhance the consistency of the mixtures. The mix design was based on the precast company specifications, which were the selection of raw materials in optimum proportions to provide concrete with the needed properties in fresh and hardened states for the performed applications, comprising 37.18 wt.% gravel, 55.76 wt.% sand, 7 wt.% OPC, and 0.06 wt.% superplasticiser additive (Code 0-0). Moreover, the design of the selected batch aimed to achieve a densely compacted cementitious matrix with good technical properties and strength.

These mixtures were then moistened by spraying with the established optimum water/cement (*w*/*c*) ratio, resulting in an effective *w*/*c* ratio of 0.45, which provided the best consistency and workability. Importantly, RAs have exhibited a higher water absorption index than NAs due to the presence of porous materials, such as mortar, ceramic, and clay. [21,22,42,43]. Thus, RAs were used under a pre-saturated condition, where the superficial pores had been partially saturated, which was an adequate method for solving the problem of the high porosity of RAs, as compared to NAs, because it helped to maintain water absorption levels during the cementation process, ensuring a water-free environment until cement hydration had occurred, as well as achieving an appropriate consistency and workability [32,36,37,42,43]. Soaking RAs in water for short intervals (approximately 3 min), in which the RAs reached up to 50% complete saturation, resulted in plastic and soft slump consistencies with a minimum loss in the compressive strength of the final concrete.

**Figure 2:**
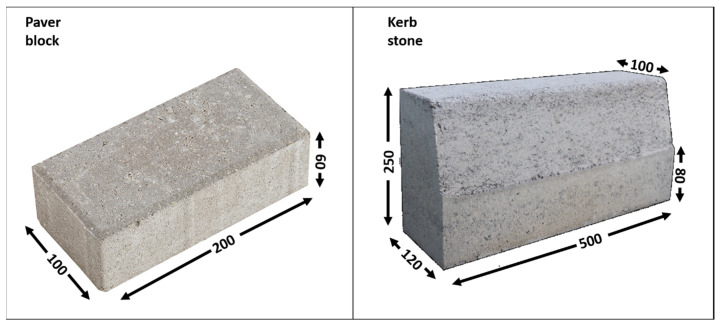
General appearance and dimensions (mm) of paver blocks (**left**) and kerb stones (**right**).

Afterwards, the mixtures were placed in steel moulds and vibrated to obtain cylindrical concrete test specimens (ϕ = 150 mm, h ≈ 300 mm), according to the UNE-EN 41166 [44] standard. Additionally, concrete paver blocks and kerb units were manufactured using hydrated mixtures. These items were placed in steel moulds provided by the precast company and compacted in duplicate by using a uniaxial hydraulic press at 30 tons, following the precast company’s requirements (Figure 2). The products were demoulded after 24 h and subsequently cured in water.

#### 2.1.3. Industrial-Scale Stage

After laboratory testing, the use of RAs from CDW as a substitute for NAs was validated at an industrial facility (Montalbán y Rodríguez S.L.). Based on the results obtained at the laboratory scale, an industrial trial was conducted using the most adequate formulations from the laboratory-tested samples.

#### 2.1.4. Experimental Methodology

The experimental plan was divided into three main stages. First, the starting materials were characterised, which included both CDW and RAs obtained after the separation method. Second, laboratory testing of concrete specimens and precast pavement units (kerbs and pavers) was performed. Finally, the industrial-scale manufacture of commercial products and validation were presented.

### 2.2. Methods

The particle size distribution of the aggregates was determined through dry analysis using a Malvern Mastersizer 3000 (Malvern, UK) laser diffraction analyser featuring two light sources, He–Ne (red) and LED (blue), and a measuring range spanning from 0.01 mm to 3500 mm. The mineral phases of the materials used were studied and identified using X-ray diffraction (XRD) in a Shimadzu (Kyoto, Japan) diffractometer model XRD 6000. Cu κα radiation, excited with 40 kV voltage and 30 mA intensity, was used. Data were recorded in the 5–60° 2θ range with a step size of 1°/min. The main elements were examined using the energy-dispersive X-ray fluorescence (EDXRF) technique in a Bruker (Billerica, MA, USA) S2 Ranger LE spectrometer fitted with a 50 W X-ray tube (50 kV, 2 mA), Pd anode, XFlash^®^ silicon drift detector with <135 eV resolution for Mn Kα and 100.000 cps and equipped with a Peltier-type cooling system (liquid nitrogen is not required) and primary filter tool changers with nine possible positions. The trace elements were measured by inductively coupled plasma-mass spectrometry (ICP-MS) in an Agilent 7500c (Santa Clara, CA, USA) by using an HP computer model HP4500^®^ (Palo Alto, CA, USA). The equipment was pre-calibrated to suitable standards.

The determination of the resistance to fragmentation of aggregates for concrete was performed using the Los Ángeles (LA) test according to the EN 1097-2 standard [45]. This test subjected a coarse aggregate sample (retained on the No. 12 (1.70 mm) sieve) to abrasion, impact, and grinding in a rotating steel drum containing a specified number of steel spheres. Once the test was complete, the calculated mass of aggregate that had broken apart into smaller sizes was expressed as a percentage of the total mass of aggregate. Therefore, lower LA abrasion loss values indicated aggregates that were tougher and more resistant to abrasion [45]. Moreover, the determination of the coefficient of friability was evaluated, which applied to the sands contained in aggregates used in the field of building and civil engineering [46]. The test involved measuring the evolution in particle size distribution of sands produced in a rotating cylinder under clearly defined conditions by fragmentation with the aid of a load in the presence of water. UNE-EN 12620:2013 [47] determined the requirements for the grading and quality of fine and coarse aggregates for use in concrete. Finally, the geometrical properties of aggregates (natural or recycled), in other words, the flakiness index of aggregates, were measured in particle sizes greater than 4 mm, or less than 80 mm, by applying the test procedure specified in UNE-EN 933-3:1997 [48].

Moreover, the physical properties, including water absorption (*WA*); specific gravity or real density (*SG*); and apparent porosity (*AP*), were calculated according to the next steps. The specimens and precast materials were immersed in water at 20 ± 5 °C for 3 days until a constant mass (immersed mass) was achieved. Before each weighing, the specimens were wiped with a moistened and squeezed cloth to remove excess water. The drying was considered complete when the surface of the concrete appeared dull (wet mass). Subsequently, each specimen was placed inside an oven at 105 ± 5 °C for 3 days until a constant mass (dry mass) was reached. WA and AP were determined based on Archimedes’ principle [49] with the following equations:

(1) $$WA\;\left(\%\right)=\frac{\left(m_{w}-m_{d}\right)}{m_{d}}\times 100$$

(2) $$AP\;(\%)=\frac{\left(m_{w}-m_{d}\right)}{{(m}_{w}-m_{i})}\times 100$$

where *m_w_* is the wet mass, *m_d_* is the dry mass, and *m_i_* is the mass immersed in water.

To calculate the real density (usually named *SG*), ISO 5018 [50] was according to the following Equation (3):

(3) $$SG\;\left(kg\;m^{-3}\right)=\frac{Powder\;Dry\;Weight}{True\;Volume}$$

where *Powder Dry Weight* is the weight (kg) of the sample milled (<62 µm) and *True Volume* is the liquid (m^−3^) displaced by the sample in a pycnometer.

Then, the impact of incorporating RAs on the resistance was evaluated in cylindrical concrete specimens with different grain sizes (CRA and FRA), according to the UNE-EN 12390-3/AC [51] standard. Then, the compressive strength (*σ*) of the test specimens was determined after 28 days by means of a hydraulic press using the following formula:

(4) σ (MPa)=PA

where *P* is the measured load at failure (N) and *A* is the resisting area (mm^2^).

The morphology and elemental composition of the concrete were analysed by scanning electron microscopy (SEM) in a HITACHI S-4800P microscope (Chiyoda, Tokyo, Japan) with an acceleration voltage of 20 kV. To analyse phase assemblages and morphology, fresh fracture surfaces were etched for 4 min in a 15% HF solution; washed ultrasonically with distilled water and ethylic alcohol; and then dried. Moreover, to analyse the porosity evolution during firing, cross-sectional samples were ground with SiC powder and subsequently polished to 1 mm with diamond pastes. Before FESEM observations, the samples were coated with Au–Pd in a BAL-TEC-Balzers SCD 050 sputter (Neugruet, Balzers, Liechtenstein).

Finally, precast concrete pavers and kerbs were evaluated according to the requirements and test methods specified in the UNE-EN 1338 [36] and UNE-EN 1340 [39] standards, respectively. The mechanical resistance of the concrete paving blocks was evaluated according to the tensile-splitting-strength (*T*) test.

(5) T MPa=0.637×k×PS

where *P* is the measured load at failure (N), *S* is the area of the failure plane (m^2^), and *k* is a correction factor (*k* = 0.87), according to the UNE-EN 1338 [38] standard.

Meanwhile, the rupture strength of the concrete kerb units was determined using the bending strength (*B*) calculated from the following equation:

(6) B (MPa)=P×L×y4×I

where *P* is the failure load (N), *L* is the distance between the supports (m), *I* is the second moment of the area determined from the work dimensions (m^4^), and *y* is the distance from the centroid to the extreme tensile fibre (m), according to UNE-EN 1340 [39].

The mechanical properties of the samples (test specimens, paver blocks, and kerb stones) were measured using an EMIC apparatus (Tokyo, Japan), DL-2000 model, at 7 and 28 days of curing and compared with the properties of standards (without RAs).

Abrasion resistance is a common quality parameter for precast concrete products such as pavers and kerbs. The test involved abrading the top surface of both materials with an abrasive material under standard temperature (298 K) and pressure (1 atm) conditions, in accordance with references [38].

The freeze–thaw resistance was evaluated in 28-day cured concrete specimens [38], using the UNE-CEN/TS 12390-9 [52] standard. Each specimen was covered with a 5 mm thick layer of freezing medium (3 wt.% NaCl) and a plastic film to maintain consistent experimental conditions (preventing evaporation and concentration changes). Then, the samples were placed in a freezing chamber at −18 to 20 °C for 24 h. During the thawing phase of the 7th, 14th, 21st, and 28th cycles, each specimen was rinsed with water into filter paper to collect the scaled material, which afterwards was dried at 105 °C for a minimum of 24 h. Freeze–thaw resistance was evaluated by measuring the mass loss per unit area.

Finally, to study the pollutant mobility in both precast materials, the EPA toxicity characteristic leaching procedure (TCLP) was performed [53]. In addition, the leachates were analysed by ICP-MS techniques.

## 3. Results and Discussion

### 3.1. Characterisation of Raw Materials

**Figure 3:**
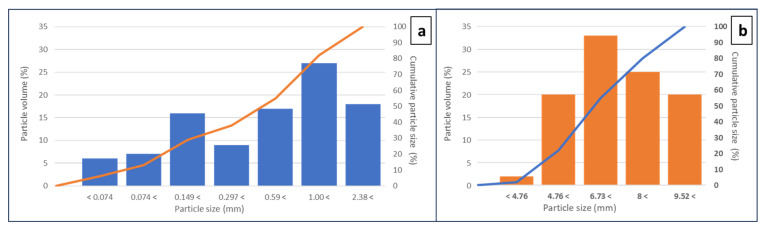
Particle size (bars) and cumulative particle size distribution (lines) of FRA (**a**) and CRA (**b**).

First, the raw materials had to be comprehensively characterised. Figure 3 depicts the particle size and cumulative particle size distribution of FRA and CRA. The results of the particle size analysis of the FRA (Figure 3a) revealed an asymmetric granulometric distribution, covering a wide range of particle sizes and displaying two main particle-size populations. The smaller population had an average diameter of approximately 149 µm; the larger population, an average size of 1 mm. In contrast, CRA exhibited a symmetrical particle size distribution, with a main population between 6.3 and 8 mm (Figure 3b). Based on the information provided in Figure 3, it was evident that in the cases of FRA and CRA, the size ratio considerably exceeded the threshold value of ρ = 1.35. This threshold represented the minimum size ratio needed for smaller particles to be accommodated within the interstitial spaces of larger particles, increasing the packing density [54]. Hence, a priori, the substitution of natural aggregates (NAs) with recycled aggregates (RAs) should not be anticipated to exert an adverse influence on particle-packing and, consequently, not on certain related properties, such as concrete porosity, water absorption, and density [40,41].

**Figure 4:**
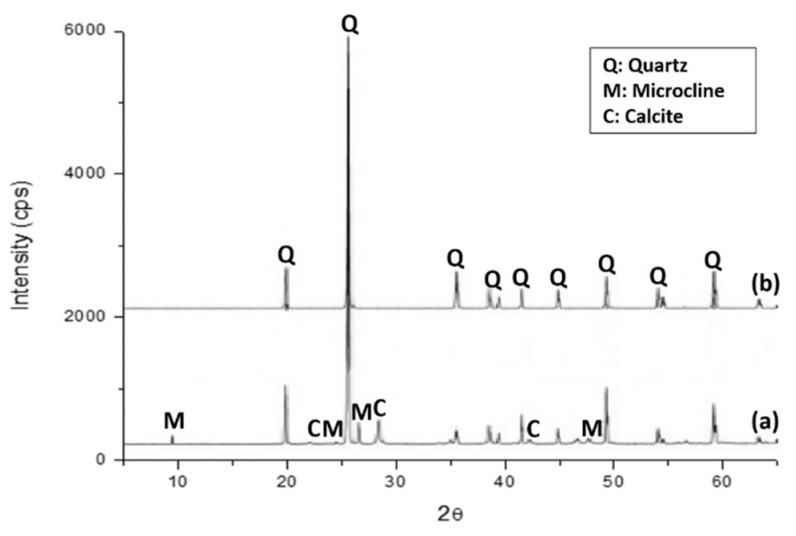
XRD pattern of natural sand (**a**) and gravel (**b**).

Second, the mineralogical compositions of NAs, natural sand (NS) (Figure 4a), and gravel (Figure 4b) were determined by XRD. The minerals detected in both samples included α-quartz (SiO_2_) and calcite (CaCO₃). Additionally, feldspar, such as microcline (KAlSi_3_O_8_), was also detected to a lesser extent in the NS.

**Figure 5:**
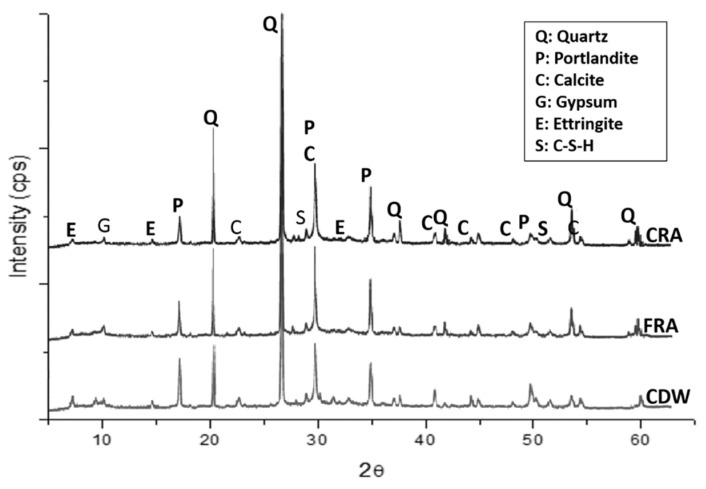
XRD pattern of CDW (**below**), FRA (**middle**) and CRA (**above**).

Furthermore, the XRD analysis of CDW and RAs revealed a complex and similar mineralogical composition among them (Figure 5). The main components were quartz, calcite, and portlandite (Ca(OH)_2_), together with other minor phases, such as calcium silicate hydrate or C-S-H (3CaO·2SiO_2_·3H_2_O); gypsum (CaSO_4_·2H_2_O); and ettringite (Ca_6_Al_2_(SO_4_)_3_(OH)_12_·26H_2_O), related to the presence of cement in CDW. However, in RAs, the peaks associated with quartz increased in intensity, while those associated with calcite and portlandite decreased, and the low-intensity peaks related to C-S-H, gypsum, and ettringite almost disappeared. This diversity of crystalline phases arose from the presence of different components, amorphous and crystalline, such as coarse gravel or crushed rocks, sand, lime, cement, and fired clay minerals [55]. Similar results were obtained in other studies on the use of CDW or natural stone waste in mortar fabrication [32,56,57].

The major elemental compositions of the materials obtained by XRF were quite similar and revealed Si, Al, Ca, Fe, Mg, and Ti as the main constituents (Table 2). These results agreed with previous studies [40,41,42,43,44]. Moreover, the presence of sulphates could lead to concrete corrosion, resulting in reduced strength and decreased durability [58]. However, the sulphate content in FRA and CRA was below 1.20%, the maximum admissible sulphate content for structural concrete in the most stringent regulation [59]. In contrast, the Portland cement type I used in this procedure was composed principally of clinker and gypsum and primarily contained CaO, SiO_2_, Al_2_O_3_, and Fe_2_O_3_ (at approximately 60, 21, 5, and 3 wt.%, respectively).

The main trace elements identified in all the samples were Ba, Zr, Sr, Zn, V, Cu, Cr, Rb, Pb, Y, and As, listed in order of decreasing abundance (Table 3). The values were of the same order of magnitude as those found in unperturbed soil [60], and consequently, the aggregates did not contain hazardous trace metals that could result in risks to the materials and human health. Portland cement could accommodate the inclusion of Cr and Zn within its matrix, resulting in alterations to its hydration characteristics. For instance, the presence of Zn delayed setting and reduced strength, while chromium induced quicker setting and enhanced early strengths. Nevertheless, due to their small quantities in RAs, it was unlikely that these trace elements would negatively impact the hydration rate of cement particles [61]. In contrast, the Na_2_O content was lower in RAs than in NAs, which could play a beneficial role since the presence of alkaline ions in the cement decreased the solubility of Ca^2+^ ions, delaying the formation of the hydrated phases.

Table 4 shows how the abrasion resistance, as determined by the angles and friability tests, of RAs (FRA and CRA) from CDW was considerably lower (25% for coarse aggregates and approximately 13% lower for fine aggregates) than that of NAs, NS, and NG. Higher values denoted a greater percentage of wear on the aggregates due to the testing procedures, which could adversely affect the mechanical properties of concrete made with RAs. According to the flakiness index, lower values (nearly 50%) were obtained for NAs, as compared to RAs. The fine content results of NG and CRA were quite similar. Nevertheless, the fine content was 64% higher in FRA than in NS. Notably, the density of NAs was higher (2–3% for BD* and 4–6% for BD**) than that of the RAs used in this study, with FRA presenting the lowest values for this property. UNE-EN12620 + A1 [47] required aggregates to have an SG greater than 2000 kg m^−3^. Consequently, both fractions, FRA and CRA, met this requirement (see Table 4). Given these results, the presence of attached mortar and ceramic materials in RAs reduced the density and increased WA, as compared to NAs. Finally, the percentage of water absorption in RAs was substantially higher (350% and 160% for fine and coarse aggregates, respectively, than in NAs, which could lead to a high demand for kneading water inside the mortar. This additional water need could affect the shrinkage of the concrete manufactured with RAs [62] because if aggregates had absorbed more of the available water in the mix, the amount of water that evaporated during the drying and curing of the concrete could be limited, thus reducing shrinkage.

### 3.2. Characterisation of Laboratory-Scale Concrete Specimens

Once the properties of the RAs had been studied, which yielded satisfactory results for their use as aggregates in concrete manufacturing, the concrete test specimens containing RAs were prepared at a laboratory scale. Table 5 lists the technological properties, including water absorption (WA), apparent porosity (AP), real density (SG), and compressive strength (σ), of these concrete specimens. Notably, in general, the addition of RAs increased WA (in this study, the increase was in the 6–70% range) and AP (between 5% and 29%), in precast concrete elements while reducing SG values (between 1% and 10% in this study). Therefore, WA and AP were directly related, especially with open porosity [63]. Additionally, the presence of unreacted cement or other porous materials could have a direct effect on cementation hydration kinetics [64], as unreacted cement is inert, does not participate in hydration, and occupies space, thus decreasing the reaction rate. Additionally, the presence of unreacted cement could reduce the release of heat during hydration, further delaying the hydration reactions.

Concerning the mechanical resistance of the concrete specimens containing RAs, σ decreased with the incorporation of FRA and especially CRA. For samples containing 75 and 100 wt.% RAs, the reduction in mechanical resistance was approximately 24%, as compared to the control specimen (0-0). This resistance reduction was likely to be affected by the observed increase in porosity in the specimens with a higher percentage of RA incorporation. In fact, the complete replacement of NAs by RAs (FRA or CRA) was accompanied by a 15% (100-0) and 29% (0-100) increase in porosity, with the resistance decreasing by 17% and 22%, respectively. The presence of ceramic materials, bonded mortar, etc., in RAs may cause an increase in porosity and the subsequent loss of mechanical resistance observed [63]. However, the replacement of up to 50 wt.% NS by FRA revealed similar strength values, with a difference of less than 5%. Generally, smaller particles dispersed the cement grains and accelerate hydration [65,66] by acting as nucleation sites and seeds for hydrated phase formation [66].

**Figure 6:**
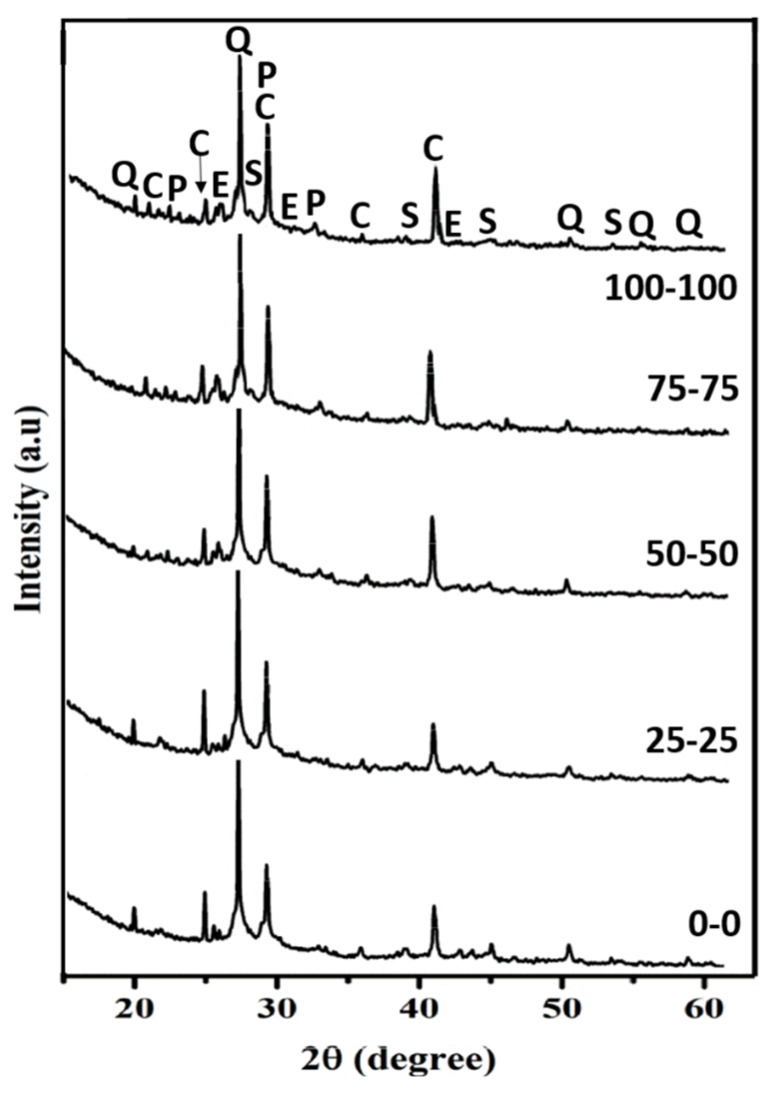
X-ray diffractograms of the test specimen including both RAs (0–100 wt.% FRA + CRA). Q: quartz; P: portlandite; C: calcite; E: ettringite; S: Calcium silicate hydrate.

Figure 6 shows the X-ray patterns of concrete specimens, including both RAs. The main crystalline phases identified were quartz, calcite, and portlandite. In addition, the low-intensity peaks may correspond to ettringite, and calcium silicate hydrate (C-S-H) was detected. Moreover, some unidentified peaks were likely associated with new crystalline phases formed during the cementing reaction with pozzolanic ceramic materials, and they should increase the resistance of the cured concrete [67]. C-S-H and portlandite, the primary products of the hydration of Portland cement, were clear examples. Subsequently, gypsum and other sulphate compounds reacted to form ettringite after mixing with water. Consequently, the incorporation of RAs increased C-S-H and reduced the intensities of the XRD peaks of quartz. NAs were mainly composed of quartz, whereas RAs also contained a considerable amount of other Ca-bearing crystalline phases found in CDW. Moreover, the intensities of calcite and portlandite slightly increased with the incorporation of RAs due to the presence of cementation products in CDW. Furthermore, carbonates, in combination with organic matter and/or free iron oxides, acted as binding agents. Calcium carbonate formed a coating on or between particles, bonding them together, sealing pores and improving the technological properties of the samples [61,67,68].

**Figure 7:**
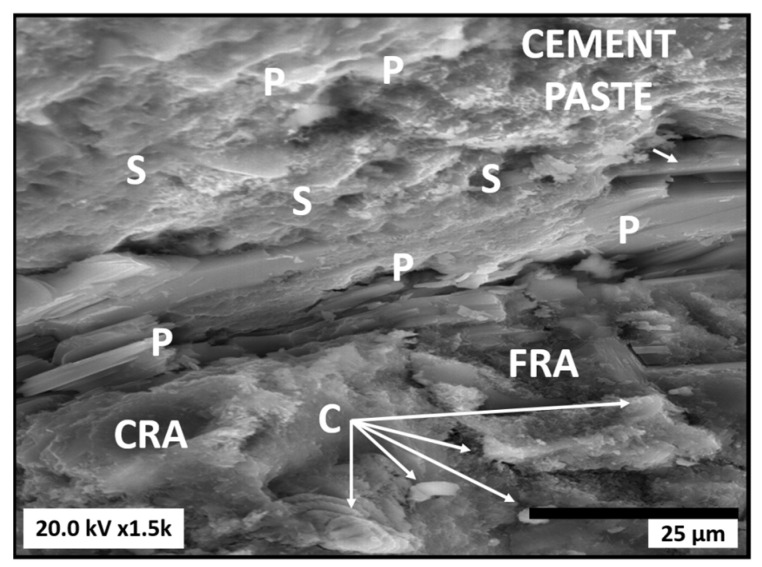
SEM observation of test specimens prepared with 100 wt.% recycled aggregates (100-100). P: portlandite; C: calcite; S: calcium silicate hydrate (C-H-S).

Figure 7 shows the microstructure of the concrete prepared with the addition of 100 wt.% of both RAs (100-100). In this case, the cement paste was visible at the top, while CRA and FRA were at the bottom. Thus, we observed that the cement paste was mainly composed of four phases: (1) surface materials (mainly C-S-H gel), (2) porous materials, (3) capillary pore space, and (4) unreacted cement. Moreover, the unreacted cement and the C-S-H gel exhibited connected pores, whereas porous materials (mainly portlandite) were generally dense polycrystalline with no connected pores. In the interfacial transition region between the cement paste and RAs, the hexagonal portlandite (P) crystals with a laminar habit and a size greater than 15 µm were clearly observed. These crystals were similar to those typically developed in conventional concrete. In addition, the C-S-H crystals were present in the surroundings of the portlandite crystals. Since these two solid phases were the primary hydration products of cement in conventional concrete, it could be concluded that the hydration behaviour of cement in concrete containing RAs remained unaffected by the replacement of NAs with RAs. Consistent with reports by Frías et al. [68], the formation of these new hydrated phases during the hydration reaction could be attributed to the presence, from the outset, of clusters with open morphologies and fairly non-uniform, porous surfaces, which enhanced their reactivity due to the presence of unreacted cement in the recycled materials (FRA and CRA). Moreover, round calcite particles smaller than 5 µm were observed on the surface of the recycled aggregate. Notably, although low-intensity peaks attributed to ettringite were identified by X-ray diffraction (Figure 6), the presence of ettringite needles was barely detectable in SEM observations. Therefore, it was expected that the problem associated with gypsum contained in recycled aggregates, which could lead to concrete expansion due to delayed ettringite formation, could be minimised [67,68].

The previous results showed that RAs obtained from CDW could be used to manufacture concrete materials with excellent physical properties.

### 3.3. Characterisation of Laboratory-Scale Precast Pavement Units

After obtaining highly favourable results with the incorporation of RAs in concrete, laboratory-scale precast pavement units (kerb units and paver blocks) containing RAs were prepared. Table 6 presents the technical property results, which revealed an increase in porosity (up to approximately 30% for paver blocks and kerb units) and water absorption (up to approximately 60% for paver blocks and 80% for kerb units), accompanied by a decrease in density (nearly 10% for paver blocks and kerb units) and resistance (up to 25% in the tensile splitting strength of paver blocks and up to 55% in the bending strength of kerb units) due to the inclusion of RAs, consistent with the findings in the previous sections. This effect could have been attributed to the lower density, higher porosity, and greater water absorption of RAs, as compared to NAs, due to the presence of adhered mortar, clay-based particles, and floating materials [69]. Furthermore, consistent with the results of similar research [70,71], the mechanical resistance was diminished because of the RA content.

According to UNE-EN 1338 [38], paving blocks were categorised into two types based on water absorption levels: Class 2 (WA < 6%, Mark B), where the paving was frost resistant, and Class 1 (WA > 6%, Mark A). Paver blocks prepared with up to 100 wt.% FRA, 75 wt.% CRA and 25 wt.% of both RAs (25-25) fell into Class 2 and Mark B, the most commercially demanded category. Additionally, higher replacement percentages were classified as Class 1 (Mark A).

Furthermore, EN-1338 [38] specified a minimum tensile-splitting-strength requirement of 3.6 MPa. Paver blocks containing up to 50 wt.% FRA or CRA and 25 wt.% FRA + CRA exceeded this minimum tensile-splitting-strength requirement. Moreover, pavers made with 25 wt.% fine (25-0) or coarse (0-25) recycled aggregates showed similar results to the control (0-0). This similarity clearly indicated that the reduction in strength was directly related to the replacement ratio, and it was tolerable if the pavers did not exceed 25 wt.%. The tensile splitting strength was mostly influenced by the characteristics of the interfacial transition zone between cement paste and aggregate (as shown in Figure 7). This reduction in strength could be attributed to the presence of a porous interlayer within the aggregate and the cement body, which could have created weak points or interfaces within the concrete mix. This result could reduce mechanical strength, as the bond between the aggregate and the cement paste may have been weaker, resulting in a reduced ability to withstand applied loads [32,71].

In contrast, kerb units manufactured with up to 75 wt.% FRA or 50 wt.% CRA or 25 wt.% FRA + CRA exhibited bending strength values exceeding the threshold of 3.5 MPa, thus meeting the requirements of Class 1 and Mark S, as established in EN-1340 [39]. Otherwise, kerbs containing 100 wt.% FRA or more than 75 wt.% CRA or 50 wt.% of both RAs did not meet the minimum requirement of bending strength greater than 3.5 MPa. According to the EN-1340 standard [39], kerb units had to at least meet the requirements of Class B, which corresponded to a WA ≤ 6%. Kerb units made with up to 75 wt.% FRA or CRA and 25% wt.% FRA + CRA showed values below this threshold. Similar results had been observed in previous studies [71,72].

The laboratory-scale study concluded that pavers and kerbs containing up to 50 wt.% FRA or CRA or 25 wt.% FRA + CRA fulfilled the requirements stated in the standards [38,39].

### 3.4. Characterisation of Industrial-Scale Precast Pavement Units

The industrial-scale results are presented in Table 7. Pavers with 25 wt.% substitution of NS by FRA presented high resistance values, even at 7 days. Furthermore, in tests conducted at 28 days, all paver elements made with RAs, except for the 25-25 combination, met the resistance requirements established in UNE-EN 1338 [38] for this type of material (at least 3.6 MPa). Regarding the water absorption results, all the paver specimens met the specifications (≤6% maximum). The control paver block (0-0) was also tested and exhibited values similar to the precast elements with RAs. These strength findings supported the results described in the preceding sections, indicating that RAs present at low replacement levels (<25 wt.%) induced insubstantial changes, as compared to the reference materials.

Regarding the abrasion wear tests (Table 7), which are one of the most common indicators of durability for this type of element, all the paver specimens met the maximum requirement of 23 mm (Class 3 and H). However, abrasion resistance values were at the tolerance limits to be classified as Class 4 and I (≤20 mm of abrasion wear). The good results in the abrasion resistance test were probably due to the higher density of the paste, as well as a stronger bond between the cement paste and the RAs [71,72,73]. Thus, a higher density generally meant less porosity in the concrete matrix. Pores in concrete could weaken the structure, making it more susceptible to abrasion. Therefore, a cement paste with a high density tended to have a higher abrasion resistance. However, the higher the bond strength was, the more difficult it was for the aggregate particles to break away from the cement paste when subjected to abrasive forces.

In terms of the freeze-thaw resistance with antifreeze salts after 28 freeze-thaw cycles, the results were similar to those of the control paver (0-0), and the values, except for 25-25, were below the threshold of ≤1 kg m^−2^ (Table 7). As already shown, the inclusion of RAs had increased the open porosity network, potentially allowing for greater mobility of the water present in the cement paste, which could help distribute the water throughout the pore network and reduce the pressure caused by the ice crystal formation [31,71].

Furthermore, results similar to those achieved with paver blocks were obtained in the kerb elements manufactured with up to 75 wt.% FRA, 50 wt.% CRA, and 25 wt.% FCA + 10 wt.% CRA (Table 7). These kerb elements satisfied the requirements stipulated by the UNE-EN 1340 [39] standard for resistance, water absorption, abrasion wear, and freeze-thaw resistance, which were >3.5 MPa, ≤2.25%, ≤23 mm, and ≤1 kg m^−2^, respectively. Moreover, UNE-EN 1338 [38] established two types of kerb units according to the mechanical resistance (B > 3.5 MPa < 5 MPa, called Class 1, and Mark S and B > 5 MPa, called Class 2 and Mark T).

Accordingly, these precast pavement elements (pavers and kerbs) made with RAs could be used in areas with very heavy pedestrian and vehicular traffic.

Finally, based on the previous results, new formulations were developed by reducing the percentage of cement in the concrete paste due to the presence of hydrated and unreacted cement in the RA fractions, which would promote cementation reactions [74]. Table 8 shows the results of the characterisation of prefabricated elements, where the cement content was reduced by up to 20 wt.%, as compared to the current amount used in the precast concrete company’s formulation. All the specimens manufactured with a 50 wt.% replacement of FRA and up to 10 wt.% of CRA, with a 7% reduction in cement content, showed, after 28 days of curing, breaking strength values above 3.6 MPa and water absorption below 6%. Regarding abrasion wear, the prefabricated product, in which only the fine aggregates had only been partially replaced, presented a value below 20 mm (Class 4), while in the specimens manufactured by replacing fine and coarse aggregates, the value was lower than 23 mm (Class 3). Therefore, these elements complied with the requirements established by the UNE-EN 1338 ANNEX F [38] standard. In contrast, a 13% reduction in cement content allowed for the replacement of only 25 wt.% of the fine aggregate fraction with recycled aggregates since higher substitution would be detrimental to mechanical resistance, resulting in values lower than those established in the standard. Finally, as shown in Table 8, the reduction in the cement content in a higher percentage was not feasible.

From the analysis of the presented results, it was concluded that the partial replacement of NAs with RAs from construction and demolition waste would be feasible. Moreover, this replacement offered the added advantage of reducing the cement content (7 wt.%) of precast concrete, resulting in considerable economic and environmental benefits.

### 3.5. Environmental Implications

The valorisation process for construction and demolition waste (CDW) conducted in this study predominantly focused on the final step from the laboratory to a real manufacturing scale. This aspect could have considerable environmental implications linked to the use of aggregates as raw materials in the production of precast concrete paving units, including paver blocks and kerb stones. In this sense, it is important to note that the production of 1 ton of gravel or sand through open-cast mining generated 0.008 tons of CO_2_ emissions, a substantial reduction from 0.001 tons of CO_2_-per-ton of aggregates (87% lower) when CDW was used [75]. Additionally, an estimated 900 kg of CO_2_ was emitted by manufacturing 1 ton of cement [76].

Given that the mixtures used in this study for the manufacture of precast concrete paving units included 37.18 wt.% gravel, 55.76 wt.% sand, 7 wt.% OPC, and 0.06 wt.% superplasticiser additive, it could be inferred that 7.43 kg of CO_2_ would be released into the atmosphere by manufacturing 1 ton of precast concrete when NAs (sand and gravel) were used. Additionally, approximately 63 kg of CO_2_ emissions could be attributed to the cement content.

Considering that the percentage of the substitution of NAs in this study had reached levels as high as 75% while still achieving acceptable results, it would be expected that the CO_2_ emissions may be reduced to as low as 2.56 kg, which represents a substantial 65% decrease in the generation of CO_2_ in the manufacturing process. Furthermore, a reduction in the cement content by as much as 13% occurred when NAs had been substituted by CDW. This led to an expected reduction of approximately 8 kg in CO_2_ emissions-per-ton in the new samples. Thus, given that CO_2_ is the main greenhouse gas, its emission could be substantially reduced by using CDW instead of NAs.

Given these findings, we could affirm that using CDW could solve a severe environmental problem by decreasing CO_2_ emissions and promoting the principles of a circular economy.

Additionally, the toxicity characteristic leaching procedure (TCLP) test was performed to assess the mobility of potential pollutant element content in the precast concrete paving. The results for all the samples analysed indicated that the concentrations of these elements were comparable to those found in the commercial reference sample, demonstrating a negligible environmental impact.

## 4. Conclusions

The results reported in this study strongly support the feasibility of producing precast concrete pavers and kerbs at an industrial scale. The main conclusions could be summarised as follows:

- Substituting natural aggregates (NAs) with recycled aggregates (RAs) did not adversely affect cement hydration but led to concretes with lower quartz content and a higher presence of Ca-bearing crystalline phases derived from mortar remains in construction and demolition waste (CDW).
- The addition of RAs increased water absorption (WA) and apparent porosity (AP) of the precast concrete elements while simultaneously decreasing density (D).
- Concrete with up to 75% NA replacement (fine or coarse) could be classified as Class 2 and Mark B, in relation to its resistance to weathering.
- Laboratory-scale pavers with RAs exceed the EN-1338 standard’s minimum-breaking-load values of 250 N mm^−1^. Moreover, a 50% replacement had no detrimental effect on the mechanical performance of the paver blocks.
- Concrete kerbs manufactured at the laboratory scale with up to 50% replacement could be classified as Class 1 and Mark S (UNE-EN 1340:2004).
- Substituting NAs with RAs at the laboratory scale enhanced the abrasion resistance for precast concrete elements, meeting the Class 3 and H standards [38,39]). However, the freeze-thaw behaviour was unaffected.
- At an industrial scale, prefabricated kerbs with up to 50% fine fraction substitution (50-0) or up to 25% and 10% fine and coarse fraction substitution (25-10) met the requirements concerning bending strength, water absorption, and abrasion wear established in UNE-EN 1340:2004 [39]. Precast paver blocks with up to 50% FRA and 20% CRA (50-20) also met UNE-EN 1338:2004 requirements [38].
- The replacement of NAs with RAs at an industrial scale enabled a reduction in cement content of up to 13 wt.%.

## Figures and Tables

**Table 1:** Compositions (wt.%) of the different mixtures studied at the laboratory scale. Each sample was labelled X-Y, where X and Y are the percentages of substitution of FRA and CRA, respectively. The percentage (%) of substitution for each fraction of FRA and CRA is indicated in parentheses.

Code	Sand	FRA	Gravel	CRA	OPC	SP *
0-0	55.76	0 (0)	37.18	0 (0)	7	0.06
25-0	41.82	13.94 (25)	37.18	0 (0)	7	0.06
50-0	27.88	27.88 (50)	37.18	0 (0)	7	0.06
75-0	13.94	41.82 (75)	37.18	0 (0)	7	0.06
100-0	0	55.76 (100)	37.18	0 (0)	7	0.06
0-25	55.76	0 (0)	27.88	9.30 (25)	7	0.06
0-50	55.76	0 (0)	18.59	18.59 (50)	7	0.06
0-75	55.76	0 (0)	9.30	27.88 (75)	7	0.06
0-100	55.76	0 (0)	0	37.18 (100)	7	0.06
25-25	41.82	13.94 (25)	27.88	9.30 (25)	7	0.06
50-50	27.88	27.88 (50)	18.59	18.59 (50)	7	0.06
75-75	13.94	41.82 (75)	9.30	27.88 (75)	7	0.06
100-100	0	55.76 (100)	0	37.18 (100)	7	0.06

* SP: superplasticiser additive.

**Table 2:** Average concentrations expressed in the oxides (n = 10) of major elements (% by weight) by XRF. Uncertainties are given as the standard deviation of the mean: *u* = (*S_x_/n*^1/2^), where *S_x_* is the standard deviation of the samples.

Raw Materials	Na_2_O	MgO	Al_2_O_3_	SiO_2_	P_2_O_5_	SO_3_	K_2_O	CaO	TiO_2_	Mn_2_O_3_	Fe_2_O_3_	LOI
CDW	0.4 ± 0.1	1.7 ± 0.3	9.8 ± 0.5	75.5 ± 7.2	0.04 ± 0.01	0.2 ± 0.1	1.0 ± 0.2	6.1 ± 0.5	1.1 ± 0.2	0.05 ± 0.02	3.2 ± 0.4	5.1 ± 0.5
CRA	0.3 ± 0.1	1.4 ± 0.2	6.8 ± 0.6	73.1 ± 6.1	0.06 ± 0.02	0.2 ± 0.1	1.2 ± 0.2	6.4 ± 0.6	0.4 ± 0.1	0.06 ± 0.02	2.3 ± 0.2	5.7 ± 0.3
FRA	<0.01	1.2 ± 0.2	5.4 ± 0.5	78.4 ± 4.9	0.06 ± 0.02	1.0 ± 0.2	0.8 ± 0.2	5.5 ± 0.4	0.2 ± 0.1	0.05 ± 0.01	1.9 ± 0.1	4.9 ± 0.4
NG	1.5± 0.3	1.4 ± 0.2	4.8 ± 0.5	63.2 ± 3.8	0.24 ± 0.08	0.6 ± 0.2	0.8 ± 0.1	10.5 ± 0.4	0.5 ± 0.1	0.05 ± 0.01	2.9 ± 0.2	1.9 ± 0.3
NS	0.6 ± 0.2	1.3 ± 0.2	3.8 ± 0.4	90.6 ± 4.2	<0.01	<0.01	0.4 ± 0.1	1.5 ± 0.3	0.4 ± 0.1	0.20 ± 0.03	1.2 ± 0.1	0.7 ± 0.1
Cement	0.2 ± 0.1	2.2 ± 0.4	5.9 ± 0.5	21.5 ± 1.4	-	2.0 ± 0.4	0.7 ± 0.1	59.8 ± 1.1	-	-	2.8 ± 0.4	3.7 ± 0.3

**Table 3:** Average concentrations (n = 10) of trace elements (mg kg^−1^) by ICP-MS. Uncertainties are given as the standard deviation of the mean: *u* = (*S_x_/n*^1/2^), where *S_x_* is the standard deviation of the samples.

Raw Materials	Ba	Zr	V	Cr	Y	Rb	Zn	Cu	Sr	Pb	As
CDW	483 ± 34	385 ± 39	81 ± 14	58 ± 12	12 ± 4	32 ± 8	88 ± 5	80 ± 4	177 ± 11	28 ± 4	3.8 ± 0.3
CRA	405 ± 49	390 ± 24	101 ± 12	90 ± 21	11 ± 2	50 ± 6	45 ± 9	81 ± 6	230 ± 16	22 ± 3	3.9 ± 0.3
FRA	529 ± 41	385 ± 31	67 ± 9	89 ± 13	22 ± 4	45 ± 4	80 ± 7	89 ± 9	301 ± 18	26 ± 3	4.0 ± 0.9
Soil (*)	584	203	97	92	21	78	67	28	348	17	4.8

* Continental crust composition [58].

**Table 4:** Average mechanical and physical properties (n = 10) of aggregates. Uncertainties are given as the standard deviation of the mean: u = (S_x_/n^1/2^), where S_x_ is the standard deviation of the samples.

Raw Materials	LA (%)	Friability	Flakiness (%)	Fines (%)	BD * (kg m^−3^)	BD ** (kg m^−3^)	SG (kg m^−3^)	WA (%)
NS	-	20.9 ± 2.8	-	4.2 ± 1.3	1450 ± 70	1660 ± 40	2450 ± 180	1.1 ± 0.2
NG	25 ± 1	-	15 ± 2	0.2 ± 0.1	1520 ± 40	1620 ± 90	2650 ± 110	1.7 ± 0.5
CDW	38 ± 5	29.5 ± 5.2	35 ± 4	12.6 ± 3.1 1.5 ± 0.4	1390 ± 180	1610 ± 190	2200 ± 340	7.1 ± 1.9
FRA	-	24.2 ± 3.5	-	6.9 ± 2.0	1410 ± 120	1590 ± 100	2280 ± 290	5.0 ± 0.9
CRA	33 ± 3	-	22 ± 4	0.3 ± 0.1	1490 ± 190	1530 ± 90	2390 ± 210	4.5 ± 1.1

* Loose condition.** Compacted condition.

**Table 5:** Technological properties (n = 10) of the different compositions of concrete test specimens prepared with RAs (after 28 days of curing). Uncertainties are given as the standard deviation of the mean: *u* = (*S_x_/n*^1/2^), where *S_x_* is the standard deviation of the samples.

Code	AP (%)	SG (g·cm^-3^)	WA (%)	σ (MPa)
0-0	8.7 ± 0.7	2.39 ± 0.22	4.8 ± 0.8	37 ± 3
25-0	9.1 ± 0.4	2.28 ± 0.20	5.1 ± 0.5	37 ± 4
50-0	9.2 ± 0.5	2.19 ± 0.18	5.7 ± 0.9	33 ± 5
75-0	9.8 ± 0.6	2.17 ± 0.23	5.8 ± 0.4	33 ± 4
100-0	10.0 ± 0.5	2.17 ± 0.20	6.1 ± 0.7	31 ± 3
0-25	9.5 ± 0.4	2.32 ± 0.10	5.0 ± 0.5	35 ± 4
0-50	9.9 ± 0.4	2.28 ± 0.24	5.4 ± 0.7	31 ± 5
0-75	10.5 ± 0.6	2.17 ± 0.19	5.9 ± 0.4	29 ± 4
0-100	11.2 ± 0.5	2.14 ± 0.22	6.5 ± 0.6	29 ± 3
25-25	9.5 ± 0.7	2.37 ± 0.25	5.9 ± 0.4	34 ± 3
50-50	10.1 ± 0.4	2.24 ± 0.15	6.9 ± 0.6	31 ± 4
75-75	10.5 ± 0.7	2.16 ± 0.31	7.3 ± 0.4	30 ± 5
100-100	11.0 ± 0.5	2.21 ± 0.21	8.2 ± 0.3	28 ± 5

**Table 6:** Technological properties (n = 10) of the paver blocks (top) and kerb units (below) manufactured with RAs (after 28 days of curing). Uncertainties are given as the standard deviation of the mean: *u* = (*S_x_/n*^1/2^), where *S_x_* is the standard deviation of the samples.

Code	AP (%)	SG (g cm^−3^)	WA (%)	T (MPa)	B (MPa)
Paver blocks					
0-0	8.7 ± 0.7	2.39 ± 0.22	5.0 ± 0.3	3.9 ± 0.2	-
25-0	9.1 ± 0.4	2.28 ± 0.20	5.2 ± 0.4	3.9 ± 0.3	-
50-0	9.2 ± 0.5	2.19 ± 0.18	5.7 ± 0.8	3.7 ± 0.2	-
75-0	9.8 ± 0.6	2.17 ± 0.23	5.8 ± 0.4	3.4 ± 0.4	-
100-0	10.0 ± 0.5	2.17 ± 0.20	6.0 ± 0.6	3.1 ± 0.4	-
0-25	9.5 ± 0.4	2.32 ± 0.10	5.2 ± 0.4	3.9 ± 0.3	-
0-50	9.9 ± 0.4	2.28 ± 0.24	5.3 ± 0.6	3.6 ± 0.2	-
0-75	10.5 ± 0.6	2.17 ± 0.19	5.8 ± 0.3	3.1 ± 0.4	-
0-100	11.2 ± 0.5	2.14 ± 0.22	6.5 ± 0.6	2.9 ± 0.4	-
25-25	9.5 ± 0.7	2.37 ± 0.25	5.9 ± 0.5	3.6 ± 0.3	-
50-50	10.1 ± 0.4	2.24 ± 0.15	6.9 ± 0.6	3.5 ± 0.3	-
75-75	10.5 ± 0.7	2.16 ± 0.31	7.2 ± 0.4	3.0 ± 0.5	-
100-100	11.0 ± 0.5	2.21 ± 0.21	8.1 ± 0.3	2.9 ± 0.6	-
Kerb units					
0-0	8.5 ± 0.5	2.37 ± 0.15	4.8 ± 0.4	-	4.9 ± 0.3
25-0	8.9 ± 0.6	2.31 ± 0.17	5.1 ± 0.4	-	4.9 ± 0.3
50-0	9.0 ± 0.6	2.22 ± 0.19	5.2 ± 0.8	-	4.6 ± 0.3
75-0	9.2 ± 0.6	2.19 ± 0.20	5.9 ± 0.7	-	3.5 ± 0.4
100-0	9.8 ± 0.5	2.17 ± 0.21	6.1 ± 0.8	-	3.3 ± 0.3
0-25	9.3 ± 0.5	2.25 ± 0.15	5.5 ± 0.5	-	4.8 ± 0.3
0-50	9.8 ± 0.6	2.25 ± 0.18	5.3 ± 0.6	-	3.5 ± 0.3
0-75	10.0 ± 0.8	2.19 ± 0.17	5.9 ± 0.9	-	2.9 ± 0.5
0-100	11.1 ± 0.6	2.15 ± 0.21	6.3 ± 0.5	-	2.2 ± 0.7
25-25	9.6 ± 0.6	2.35 ± 0.22	5.8 ± 0.8	-	3.8 ± 0.4
50-50	10.2 ± 0.7	2.29 ± 0.17	6.4 ± 0.6	-	3.3 ± 0.3
75-75	10.9 ± 0.8	2.18 ± 0.21	7.9 ± 0.4	-	2.8 ± 0.4
100-100	11.1 ± 0.8	2.19 ± 0.25	8.7 ± 0.8	-	2.4 ± 0.5

**Table 7:** Technological properties (n = 50) of paver (top) and kerb (below) elements prepared with RAs (the results show the average values of four measurements). Uncertainties are given as the standard deviation of the mean: *u* = (*S_x_/n*^1/2^), where *S_x_* is the standard deviation of the samples.

	Mechanical Strength (MPa)		WA (%)	Abrasion Wear (mm)	Freeze-Thaw Cycles (kg m^−2^)
	7 Days	28 Days	28 Days	28 Days	28 Days
Paver blocks					
0-0	4.2 ± 0.2	5.1 ± 0.2	4.1 ± 0.2	20.0 ± 0.3	0.89 ± 0.50
25-0	4.0 ± 0.3	4.8 ± 0.5	3.7 ± 0.3	20.1 ± 0.1	0.90 ± 0.69
50-0	3.2 ± 0.3	4.1 ± 0.3	3.8 ± 0.5	20.0 ± 0.1	0.90 ± 0.58
0-25	2.7 ± 0.2	4.3 ± 0.3	4.3 ± 0.4	20.4 ± 0.3	0.91 ± 0.10
0-50	2.9 ± 0.5	3.9 ± 0.4	3.9 ± 0.2	20.2 ± 0.4	0.98 ± 0.09
25-10	2.6 ± 0.3	3.9 ± 0.2	4.0 ± 0.2	20.3 ± 0.4	1.00 ± 0.12
25-25	2.6 ± 0.3	3.5 ± 0.2	4.5 ± 0.3	20.3 ± 0.4	1.05 ± 0.15
Requirements *	-	3.6	6	23	1
Kerb units					
0-0	4.2 ± 0.2	5.9 ± 0.2	4.1 ± 0.2	20.0 ± 0.3	-
50-0	3.9 ± 0.2	5.3 ± 0.3	4.6 ± 0.3	20.1 ± 0.4	-
75-0	3.0 ± 0.4	3.8 ± 0.3	4.9 ± 0.3	20.4 ± 0.4	-
0-25	3.9 ± 0.4	5.4 ± 0.4	3.9 ± 0.4	20.2 ± 0.4	-
0-50	2.8 ± 0.5	3.7 ± 0.3	4.1 ± 0.2	20.5 ± 0.4	-
25-10	3.6 ± 0.3	5.1 ± 0.4	4.4 ± 0.4	20.3 ± 0.4	-
25-25	3.1 ± 0.3	3.4 ± 0.5	4.5 ± 0.3	20.3 ± 0.4	-
Requirements **	-	5	6	23	-

* UNE-EN 1338 [38]. ** UNE-EN 1340 [39].

**Table 8:** Technological properties (n = 50) of paver elements prepared with RAs (the results show the average values of four measurements). Uncertainties are given as the standard deviation of the mean: *u* = (*S_x_/n*^1/2^), where *S_x_* is the standard deviation of the samples.

	Cement Reduction (wt.%)	Breaking Strength (MPa)		WA (%)	Abrasion Wear (mm)
		7 Days	28 Days	28 Days	28 Days
0-0	-	4.2 ± 0.2	5.1 ± 0.2	4.1 ± 0.2	20.0 ± 0.3
25-0	20	2.6 ± 0.2	2.7 ± 0.2	3.6 ± 0.9	19.9 ± 0.1
25-0	13	2.9 ± 0.2	3.6 ± 0.2	4.3 ± 0.2	20.5 ± 0.5
50-0	20	1.7 ± 0.1	1.9 ± 0.1	3.9 ± 0.8	20.0 ± 0.6
50-0	13	2.2 ± 0.2	2.3 ±0.2	3.9 ± 0.3	20.4 ± 0.1
50-0	7	3.3 ± 0.1	3.7 ± 0.1	4.2 ± 0.2	19.9 ± 0.4
50-10	7	3.6 ± 0.1	3.8 ± 0.1	3.7 ± 0.6	20.1 ± 0.3
50-20	7	2.6 ± 0.2	3.1 ± 0.2	3.4 ± 0.2	20.4 ± 0.6
Requirements *	-	-	3.6	6	20

* UNE-EN 1338 [38].

## Data Availability

Data are contained within the article.
